# The effect of patient positions on perfusion index

**DOI:** 10.1186/s12871-018-0571-z

**Published:** 2018-08-17

**Authors:** Hakan Tapar, Serkan Karaman, Serkan Dogru, Tugba Karaman, Aynur Sahin, Gulsen Genc Tapar, Fatih Altiparmak, Mustafa Suren

**Affiliations:** 1 Department of Anesthesiology and Reanimation, Gaziosmanpasa University, Faculty of Medicine, 60100 Tokat, Turkey; 2 Tokat State Hospital, Cardiology Clinic, Tokat, Turkey

**Keywords:** Perfusion, Surgery, Position

## Abstract

**Background:**

The optimal position for surgery is one in which the patient is provided the best possible surgical intervention and put at minimum risk. Different surgical positions may cause changes in tissue perfusion. This study investigates the relationship between surgical patient positions and perfusion index.

**Methods:**

A sample of 61 healthy individuals with no peripheral circulatory disorders, chronic diseases, or anemia was included in this study. Participants held six different positions: supine, prone, 45-degree sitting-supine, 45-degree supine with legs lifted, Trendelenburg (45-degrees head down), and reverse Trendelenburg (45-degrees head up). Perfusion index values were then measured and recorded after individuals held their positions for five minutes.

**Results:**

Participants’ perfusion index values were affected by different body positions (*p* < 0.05). Perfusion index was lowest in the sitting position (4.5 ± 2.5) and highest in individuals with Trendelenburg position (7.8 ± 3.8).

**Conclusion:**

Different body positions can cause changes in tissue perfusion. This should be considered in patient follow-up along with the perfusion index.

## Background

Anesthesia team should guarantee the patient safety first. To achieve this, it is necessary to be aware of both the anesthetic methods and all potential perioperative physiological changes [1]. According to the surgeon’s preference, patients put in supine, prone, sitting, lithotomy, Trendelenburg or reverse Trendelenburg position. Different hemodynamic and respiratory changes take place depending on a patient’s surgical position, making it important to know the possible physiological effects of each position [2]. Bleeding and fluid flux (from extracellular space to intravascular) may also occur during surgery. It can disrupt tissue perfusion in cases of advanced conditions. Perfusion index (PI) is used for peripheral tissue monitorization [3].

Global tissue perfusion can be evaluated both clinically (by evaluating skin coldness, paleness, mottling, and prolongation of capillary retention time) and biochemically (using serum lactate and central venous oxygen saturation). The most appropriate method for tissue perfusion should be non-invasive, quick, and easily measurable [4]. PI is a non-invasive method and is the non-pulsatile flow rate of the pulsatile flow [5]. PI works by measuring changes in finger peripheral perfusion through a pulse oximeter [6].

PI is a rapid indicator of microcirculatory changes and may help anesthetists to see the disturbances in circulation [7–9]. PI is used to evaluate hypoperfusion caused by bleeding and fluid loss in patients undergoing surgery, however there has been no study existed in the literature to compare the effects of various patient positions on PI. The findings of this study contribute to our understanding of the PI values during surgery. Therefore, the aim of this study is to evaluate whether the PI was influenced by changes in several surgical body positions.

## Methods

The study was approved by the local ethics committee of Gaziosmanpasa University (17-KAEK-010), and the presented data are a subset of a previously registered study at clinicaltrials.gov (Grant number: NCT03121443). This prospective observational study involved 61 healthy volunteers. Participants were excluded if they were older than 45 or younger than 18 or if they had peripheral circulatory disorders, chronic diseases, body mass index (BMI) > 30, smoking habits, or anemia. Participants who took part in the study had no more than 2–3 h of fasting period.

All participants gave informed written consent prior to the study. Participants were taken to the recovery room, where they waited 15 min before measurements were taken.

The age, weight, height, and cigarette history of participants, along with room temperature, were recorded. After monitoring PI (Masimo Radical 7; Masimo Corp., Irvine, CA, USA) using the right ring finger, each participant first took the supine position. The new perfusion index value was obtained after five minutes. Second, the patient’s position was changed to 45-degree back-up sitting position, and the PI measurement was repeated after five minutes. The participant were then taken to Trendelenburg (45-degrees head down), reverse Trendelenburg (45-degrees head up), prone, and 45-degree legs-lifted supine positions, respectively. Before each position change, participants were taken to the supine position and the new position was assigned after five minutes of rest. Participants held each position for five minutes to stabilize the circulatory condition of the participant before measurements were taken [10]. Each measurement was performed by the same researcher. After two minutes of each position, PI, blood pressure, heart rate, and SpO2 values were recorded.

### Statistical analysis

A pilot study revealed a PI mean of 2.79 ± 1.93 for supine position. Using this value, and a 25% increase in this value for each position (accepting type I error of 0.05 and a power of 0.80), a total of 61 patients were required to find a statistically significant difference between each position.

The descriptive characteristics were expressed as numbers and percentages in the categorical variables and as means, standard deviations, and medians. The Kolmogorov-Smirnov test was employed to test the normality of the data’s distribution. Measurements were compared with repeated measures ANOVA test. The chi square test was used to compare categorical data. Data analysis was performed with the IBM SPSS version 22.0 statistical package (Chicago, IL, USA). A *p* value < 0.05 was considered statistically significant.

## Results

The demogragraphic characteristics of the participants were presented in Table 1. Comparison of hemodynamic changes in different body positions was showed in Table 2.

**Table 1:** Distribution of the sample in terms of demographics

Characteristics	n	Mean ± SD
Gender (M/F)	18/43	
Age (year)		30.5±5.5
BMI		25.2±4,4
Height (cm)		172.3±8.5
Room temperature (°C)		22.7±1.2

*BMI* Body mass index, *M* male, *F* female

**Table 2:** Comparison of hemodynamic changes in different body positions

Body Position	Pulse Rate	Systolic blood pressure	Diastolic blood pressure	SpO2
S	72.3±8.8	114.8±10.2	64.3±6.6	97.0±1.0
T	71.5±8.1	113.4±12.9	59.8±14.4	96.7±1.4
LL	71.9±9.6	111.7±9.8	63.2±9.0	96.5±1.5
P	73.1±8.9	110.5±11.0	63.9±7.6	96.2±1.4
RT	72.3±8.9	113.1±12.2	65.6±7.8	96.7±1.4
SS	75.6±9.4	114.3±10.2	66.6±10.8	96.6±1.4

*S* Supine, *T* Trendelenburg, *LL* 45-degree supine with legs lifted, *P* Prone, *RT* Revers Trende-lenburg, *SS* 45-degree sitting-supine

**Fig. 1:**
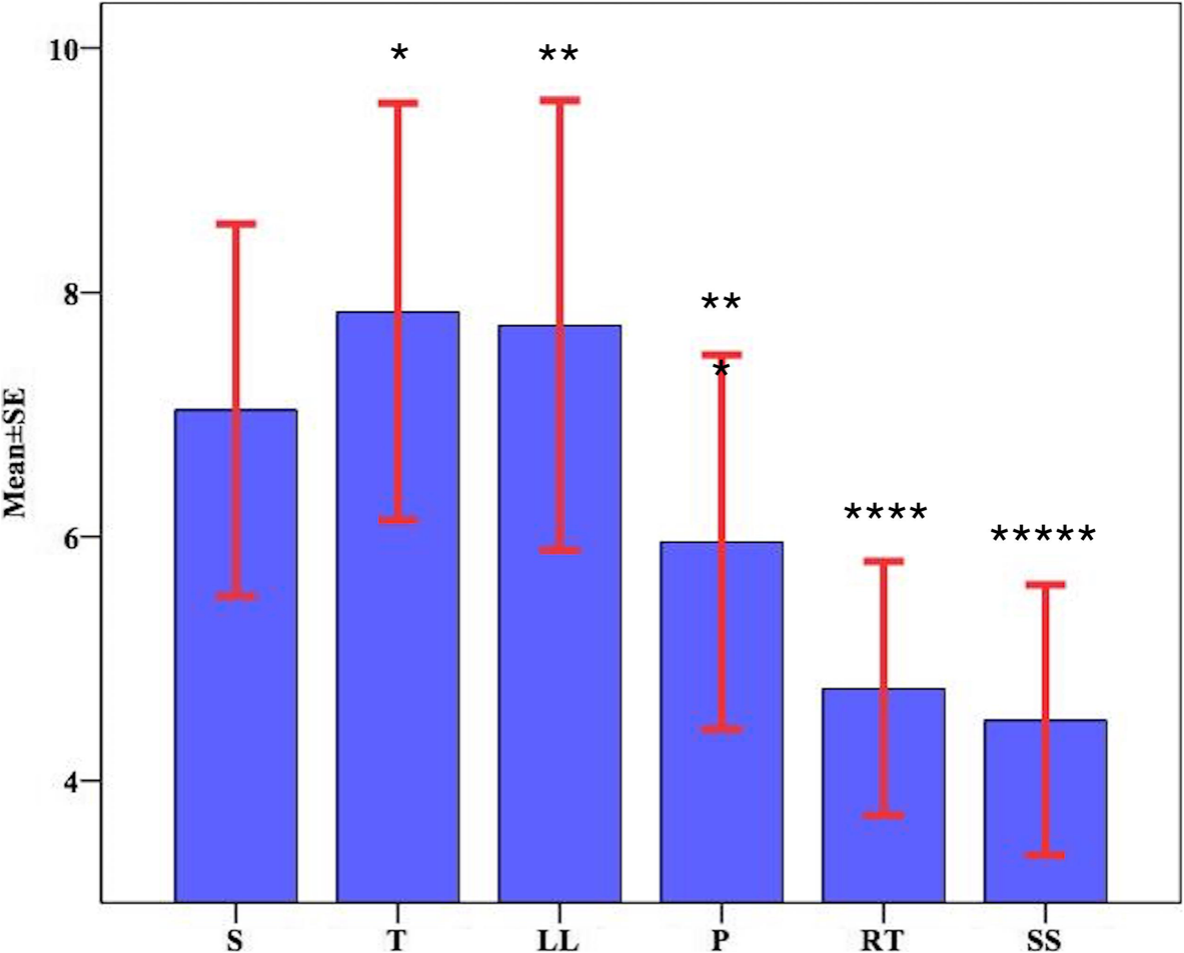
Comparison of perfusion index mean values in different body positions. S, Supine; T,Trendelenburg; LL, 45-degree supine with legs lifted; P, Prone; RT, Revers Trendelenburg; SS, 45-degree sitting-supine. Repeated measures ANOVA. The following comparisons are found statistically significant: ^*^S-T: *p* = 0.003; ^**^S-LL: *p* = 0.041; ^***^S-P: *p* = 0.007; ^****^S-RT: *p* = < 0.001; ^*****^S-SS: *p* = < 0.001

Different mean perfusion index values were measured for each position. The values for the supine, Trendelenburg, reverse Trendelenburg, 45-degree back-up sitting position, 45-degree legs-lifted supine, and prone positions were 7.0 ± 3.4, 7.8 ± 3.8, 4.8 ± 2.3, 4.5 ± 2.5, 7.7 ± 4.2, and 6.0. ± 3.4, respectively. The highest PI value was measured in the Trendelenburg position (7.8 ± 3.8) and the lowest value was found in the 45-degree back-up sitting position (5.0 ± 2.5). There was a statistically significant difference in the PI values measured while the individuals were in the different positions as compared to the supine position (Fig. 1).

## Discussion

This study showed that PI values among healthy individuals varied according to body position (using the supine position measurements as a baseline). However, no such relationship was found between surgical patient positions and systolic blood pressure, diastolic blood pressure, or pulse rate.

Cardiac output (CO) is the only parameter that affects both oxygen delivery and blood pressure, and the treatment and evaluation of patients is based largely on the optimization of this parameter [11]. CO is determined by the amount of blood pumped from the heart in approximately one minute (5 L/min) and systemic vascular resistance (SVR) to heart rate (HR) (CO:SVR × HR). A decrease in the preload and contractility of the heart causes a decrease in CO, in response to which the body increases cardiac contractility, SVR, and HR through baroreceptors to increase CO. This situation results in clinically cold and weak peripherals.

In circulatory disorders associated with hypovolemia and low CO, blood from vital organs (e.g., the brain and heart) is replaced by blood from non-vital organs (such as the skin). Perfusion impairment in non-vital organs begins earlier and resolves later [4]. Pulsatile flow (arterial) governing PI is affected by vasoconstriction and vasodilation [12]. Redistribution of blood causes vasoconstriction, resulting in decreased PI values.

Sitting position can cause blood pressure fluctuations and health-threatening complications during surgery [13, 14]. Smith et al. report that in conscious volunteers, a head-up tilt causes both an immediate and stabilized response, the immediate period being the first few seconds and the stabilized period being between 30 s and 20 min [15]. The stabilization period saw a 30–40% increase in total vascular resistance, decrease an approximately 25–30% decrease in thoracic blood volume, and a 15–30% in CO. In a study analyzing cardiac index, pulmonary, and total blood volume values with the thermodilution technique, it was observed that these values decreased in sitting position. This phenomenon is connected to the passage of blood from the intra-thoracic area to the extra-thoracic area [16]. When subjects changed from the supine position to the 45-degree sitting position, their perfusion indices decreased significantly. This may be associated with lowered cardiac output and increased vascular resistance as compensatory mechanisms.

The hemodynamic changes seen in the reverse Trendelenburg position are similar to those observed in sitting position [17]. The sympathetic nervous system is activated by baroreceptors to prevent hypotension in awake patients, which causes an increase in SVR to protect blood pressure [18]. Changing from the supine position to the 45-degree reverse Trendelenburg (45-degree head-up) decreases the PI significantly. This can be explained by the decrease in venous return and increase in systemic vascular resistance.

The prone position causes significant changes in the cardiovascular system, including decreases in arterial blood pressure, CO, and cardiac index [1]. The reason for this decrease is the high thoracic pressure the position creates, which affects arterial filling and left ventricular compliance [18]. Another study comparing the changes in cardiac index between prone and supine position showed that prone position was lowered the cardiac index by 24%. However, there was no change in patient blood pressure [19]. Our study also found no significant change in blood pressure from the supine position to the prone position.

The 45-degree legs-lifted supine position increases venous return and preload, causing a sudden increase in cardiac output [11]. Keller et al. tested changes in preload induced by passive leg raising (PLR) in spontaneously breathing volunteers. Pleth Variability Index (PVI) decreased and CO increased during changes in body position, but systolic blood pressure, diastolic blood pressure, and HR did not change. Keller et al. state that these changes occur maximally during the first minute and disappear after a few minutes [20]. In our study, the perfusion index was decreased by changing the position from 45-degree legs-lifted to supine.

The use of healthy volunteers in this study showed that surgical patient position affects the perfusion index. The highest perfusion index values were observed in the Trendelenburg position. Positional PI changes are usually due to compensatory response factors affecting CO (reduction in venous conversion or venous compression) and increasing systemic vascular resistance.

This prospective study has one limitation. The findings obtained in this study were included only parameters measured from healthy individuals. Therefore, it can be more accurate to conduct the study on surgical patients to increase the generalizability of the outcomes.

## Conclusions

The present study revealed that PI changes according to body position in which has the highest value during Trendelenburg position and the lowest during 45-degree sitting supine. This finding can be important in surgical patients whose position might change during surgery thus affecting the PI value and requires a new baseline to follow-up the PI. This study contribute to our understanding of the PI.
